# Phosphoinositide 3-Kinaseγ Controls the Intracellular Localization of CpG to Limit DNA-PKcs-Dependent IL-10 Production in Macrophages

**DOI:** 10.1371/journal.pone.0026836

**Published:** 2011-10-28

**Authors:** Kaoru Hazeki, Yukiko Kametani, Hiroki Murakami, Masami Uehara, Yuki Ishikawa, Kiyomi Nigorikawa, Shunsuke Takasuga, Takehiko Sasaki, Tsukasa Seya, Misako Matsumoto, Osamu Hazeki

**Affiliations:** 1 Division of Molecular Medical Science, Graduate School of Biomedical Sciences, Hiroshima University, Hiroshima, Japan; 2 Department of Pathology and Immunology, Akita University School of Medicine, Akita, Japan; 3 Department of Microbiology and Immunology, Hokkaido University Graduate School of Medicine, Sapporo, Japan; University of Kansas Medical Center, United States of America

## Abstract

Synthetic oligodeoxynucleotides containing unmethylated CpG motifs (CpG) stimulate innate immune responses. Phosphoinositide 3-kinase (PI3K) has been implicated in CpG-induced immune activation; however, its precise role has not yet been clarified. CpG-induced production of IL-10 was dramatically increased in macrophages deficient in PI3Kγ (p110γ^−/−^). By contrast, LPS-induced production of IL-10 was unchanged in the cells. CpG-induced, but not LPS-induced, IL-10 production was almost completely abolished in SCID mice having mutations in DNA-dependent protein kinase catalytic subunit (DNA-PKcs). Furthermore, wortmannin, an inhibitor of DNA-PKcs, completely inhibited CpG-induced IL-10 production, both in wild type and p110γ^−/−^ cells. Microscopic analyses revealed that CpG preferentially localized with DNA-PKcs in p110γ^−/−^ cells than in wild type cells. In addition, CpG was preferentially co-localized with the acidic lysosomal marker, LysoTracker, in p110γ^−/−^ cells, and with an early endosome marker, EEA1, in wild type cells. Over-expression of p110γ in Cos7 cells resulted in decreased acidification of CpG containing endosome. A similar effect was reproduced using kinase-dead mutants, but not with a ras-binding site mutant, of p110γ. Thus, it is likely that p110γ, in a manner independent of its kinase activity, inhibits the acidification of CpG-containing endosomes. It is considered that increased acidification of CpG-containing endosomes in p110γ^−/−^ cells enforces endosomal escape of CpG, which results in increased association of CpG with DNA-PKcs to up-regulate IL-10 production in macrophages.

## Introduction

Oligodeoxynucleotides containing unmethylated CpG motifs (CpG) are powerful immune adjuvants that induce the production of cytokines, including IL-6, IL-10, IL-12, IFN-α/β, and TNF-α [1], [2]. Although previous studies have established that CpG-induced immune responses are mediated by endosomal TLR9 [3]–[5], cytoplasmic DNA-PKcs are also involved in CpG-signaling independent of TLR9 [6], [7]. Thus, intracellular trafficking of CpG is critical to select downstream signaling molecules, which determine the cytokine species produced by macrophages [7], [8].

Phosphoinositide 3-kinase (PI3K) has been reported to be both a positive and negative regulator of CpG-mediated cytokine production. CpG-induced IL-12 production is increased in plasmacytoid dendritic cells (pDC) from p85α^−/−^ mice, and by treatment of wild-type pDC with wortmannin [9]. Likewise, CpG-induced iNOS expression is increased by treating RAW264.7 cells with wortmannin [10]. By contrast, another group has reported that wortmannin inhibits CpG-induced production of IL-12, IL-6, TNF-α, and NO from RAW264.7 cells [11]. This inhibition has been considered to be the result of wortmannin-mediated disruption of class III PI3K signaling, which is responsible for CpG uptake [11]. Similarly, wortmannin inhibits CpG-induced IL-12 production by inhibiting CpG internalization in mouse-derived bone marrow cells [12]. In human pDC, another PI3K inhibitor, LY294002, also inhibits CpG-induced type I IFN production [13]. In this case, the uptake and endosomal trafficking of CpG are not affected, but nuclear translocation of IRF-7 was inhibited by LY294002, and also, by a specific inhibitor of PI3Kδ, IC87114 [13]. In addition, the PI3K/mTOR/p70S6K pathway plays a substantial role in the spatial interaction of TLR9/MyD88/IRF7, which is indispensable for the induction of type I IFN production by pDC [14]. These reports have indicated that PI3Ks play some roles in trafficking of CpG itself or its downstream molecules.

Pan- and/or some other specific PI3K inhibitors were used in all of the previous studies described above. All of these inhibitors bind competitively to the ATP binding pocket of PI3Ks and block kinase activity. Since DNA-dependent protein kinase catalytic subunit (DNA-PKcs) shares similar ATP binding site as a member of the PI3K-like kinase family, these inhibitors, even isoform-specific inhibitors, more or less inhibit DNA-PKcs [15]. This makes it difficult to elucidate the precise role of DNA-PKcs and PI3Ks in CpG-mediated cytokine production. In this paper, we used class IB PI3K (p110γ) knockout mice and SCID mice having mutations in DNA-PKcs to estimate their roles in CpG-mediated cytokine production. In agreement with a current report, DNA-PKcs play a substantial role in CpG-mediated IL-10 production in macrophages [7]. By contrast, p110γ specifically down-regulates IL-10 production following CpG-stimulation. Quantitative analysis of microscopic images showed that CpG localized preferentially with DNA-PKcs in the cytosol in p110γ^−/−^ cells to a greater extent than in wild-type cells. We propose a novel regulatory role of p110γ in CpG-induced production of IL-10 through modulation of the intracellular trafficking of CpG.

## Results

### p110γ deficiency specifically increased IL-10 production upon CpG stimulation in macrophages

**Figure 1:**
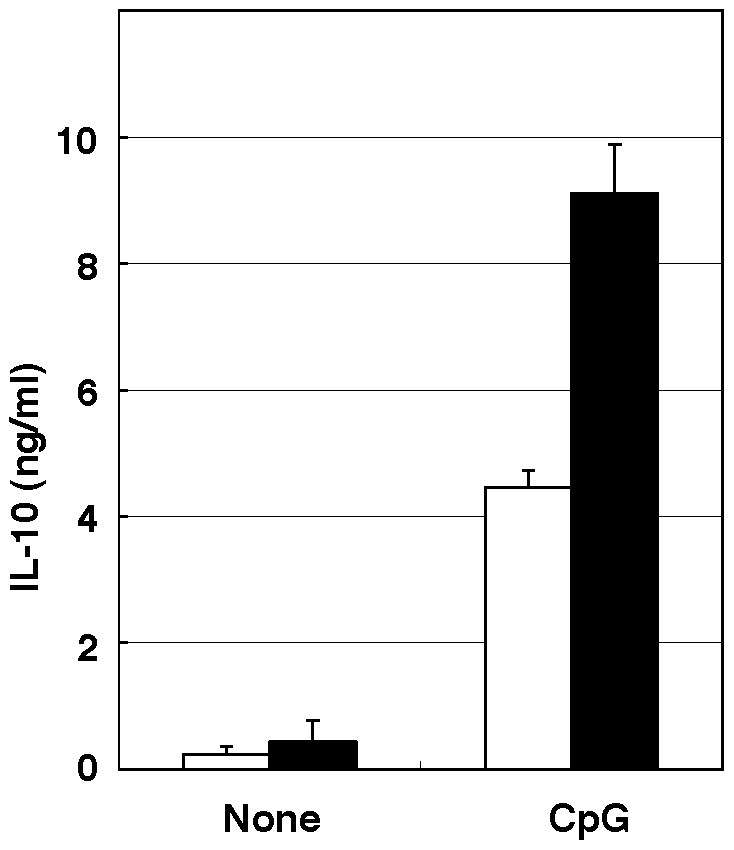
Increased IL-10 production following CpG stimulation of p110γ^−/−^ cells. Macrophages from wild type (open bar) or p110γ^/−^ mice (solid bar) were incubated in 24-well plates with 10 ng/mL LPS, 200 ng/mL CpG, 50 µg/mL polyI:C or 200 nM Malp2 for 18 h. The amount of IL-10 in the medium was determined by ELISA. The values are the means ± SD of duplicate cultures from three independent experiments.

Mouse macrophages generated IL-10 in response to CpG (Fig. 1). Since PI3K has been implicated in the regulation of TLR-induced IL-10 production [16], we tested the effect of p110γ depletion on IL-10 production. CpG-induced IL-10 production was dramatically increased in macrophages from p110γ^−/−^ mouse (Fig. 1). Although IL-10 production sometimes varied extremely between experiments, IL-10 production in wild type mice was always approximately half of that in p110γ^−/−^ mice in each paired experiment. We also tested the cytokine production using macrophages from p85α^−/−^ mice, and from p110δ^KD/KD^ mice; neither displayed CpG-specific changes in IL-10 production similar to what was seen with p110γ^−/−^ cells (data not shown).

### Wortmannin inhibited IL-10 production induced by CpG, but increased that induced by LPS

**Figure 2:**
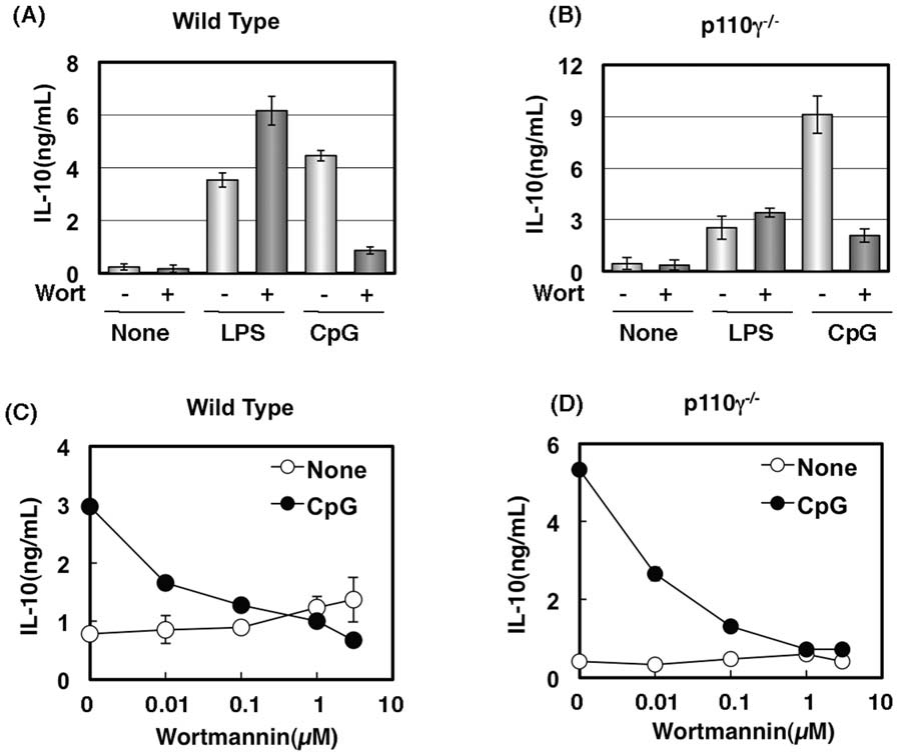
Inhibition of CpG-induced IL-10 production by wortmannin. Macrophages from wild type (A, C) or p110γ^−/−^ mice (B, D) were pre-incubated with 0.1 µM (+ in A, B), or increasing concentration of wortmannin (C, D) for 15 min, followed by the addition of 10 ng/mL LPS (A, B) or 200 ng/mL CpG (A–D), for 18 h. The amount of IL-10 in the medium was determined by ELISA. The values are the means ± SD of duplicate cultures from three independent experiments.

Macrophages were treated with a pan-PI3K inhibitor, wortmannin. CpG-induced IL-10 production was almost completely inhibited by wortmannin while LPS-induced IL-10 production was rather increased in wild type cells (Fig. 2A). The effect of wortmannin on LPS-induced IL-10 production may be the result of the inhibition of p110γ, because the augmentation was not observed in p110γ^−/−^ cells (Fig. 2B). However, wortmannin inhibited severely CpG-induced IL-10 production in p110γ^−/−^ cells, as well as in wild type cells (Fig. 2C. D). The inhibition by wortmannin was specific to IL-10 production, because IL-12 production was unchanged by the treatment (data not shown). These data indicate that the target molecule of wortmannin responsible for the suppression of CpG-induced IL-10 was not p110γ.

### p110γ-mediated negative regulation of IL-10 production was independent of kinase activity

**Figure 3:**
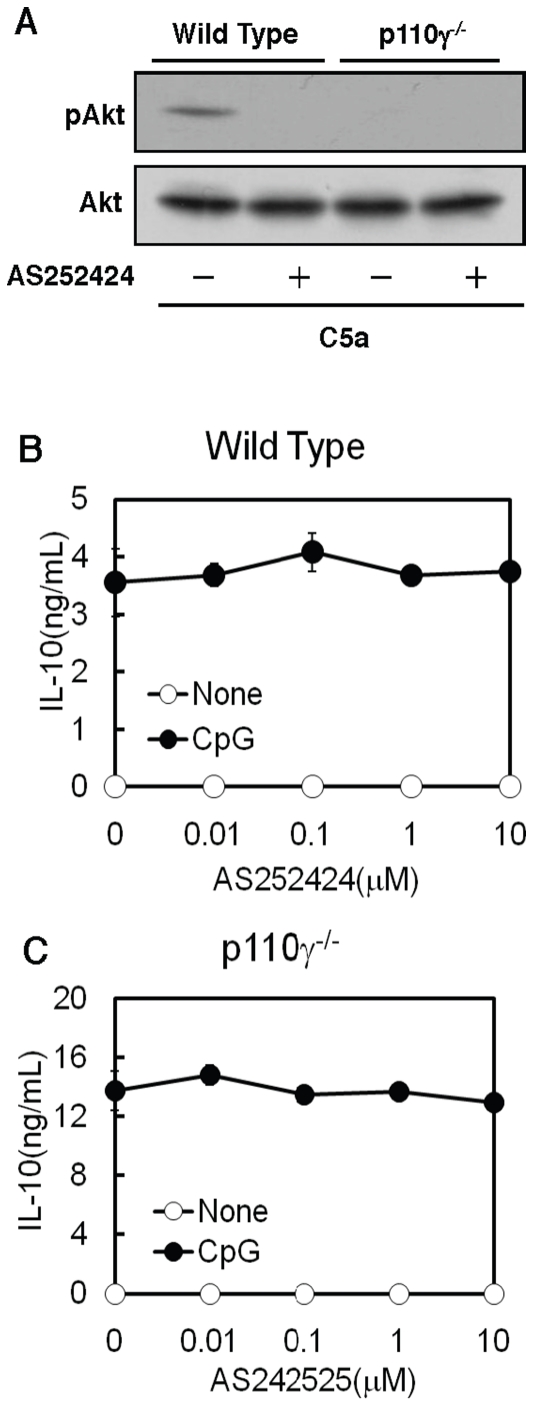
Irrelevance of the kinase activity of p110γ in the regulation of IL-10 production. (A) Macrophages from wild type or p110γ^−/−^ mice were pre-incubated with 10 µM AS252424 for 15 min, followed by the addition of 100 ng/mL C5a for 5 min. Total cell lysates from the treated cells were analyzed by Western blot. (B and C) Macrophages from wild type (B) or p110γ^−/−^ mice (C) were pre-incubated with increasing concentrations of AS252424 for 15 min, followed by the addition of 200 ng/mL CpG for 18 h. The amount of IL-10 in the medium was determined by ELISA. The values are the means ± SD of duplicate cultures from three independent experiments.

Since a pan-PI3K inhibitor, wortmannin, did not mimic the effect of p110γ deficiency on CpG-induced IL-10 production, we next tested the effect of the p110γ-specific inhibitor, AS252424, on CpG-induced IL-10 production. C5a-induced Akt phosphorylation was completely abolished in p110γ^−/−^ cells (Fig. 3A) [17]. The result confirmed that the C5a action is dependent on p110γ. AS252424 inhibited the C5a-induced Akt phosphorylation (Fig. 3A), indicating that the compound is a powerful tool for investigating the role of p110γ. Surprisingly, AS252424 did not cause an increase in CpG-induced IL-10 production in the wild type cells nor in p110γ^−/−^ cells (Fig. 2B, C). These data suggest that the negative regulation of CpG-induced IL-10 production by p110γ was not dependent on its kinase activity.

### CpG-induced IL-10 production was abolished in SCID mice

**Figure 4:**
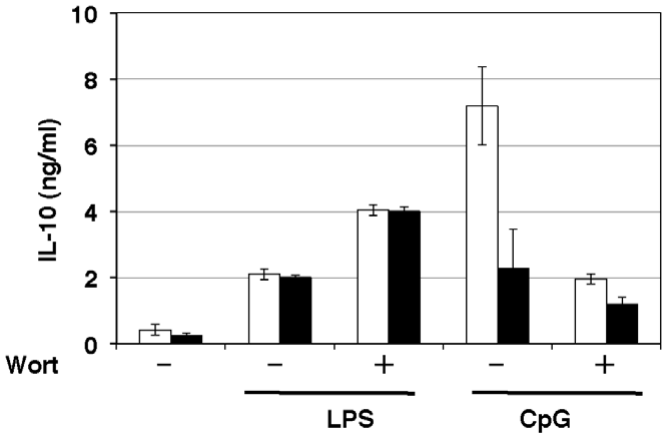
Failure of CpG to stimulate IL-10 production in SCID mice. Macrophages from wild type (open bar) or SCID (solid bar) mice were preincubated with 0.1 µM wortmannin (+) or vehicle (−) for 15 min, followed by the addition of 10 ng/mL LPS or 200 ng/mL CpG, for 18 h. The amount of IL-10 in the medium was determined by ELISA. The values are the means ± SD of duplicate cultures from three independent experiments.

Recently, an indispensable role of DNA-PKcs in CpG-induced IL-10 production has been reported [7]. Since DNA-PKcs has a PI3K-like catalytic domain, the kinase activity is susceptible to wortmannin. As reported earlier [7], CpG-induced IL-10 production was defective in SCID mice (Fig. 4). Additionally, wortmannin inhibited significantly CpG-induced IL-10 production in wild type, whereas it did not affect this process in SCID mice (Fig. 4). This result suggests that wortmannin suppressed CpG-induced IL-10 production through inhibition of DNA-PKcs. By contrast, LPS-induced IL-10 production in SCID was identical to wild type with the same background mice (Fig. 4).

### Co-localization of DNA-PKcs and CpG was increased in p110γ^−/−^ cells

**Figure 5:**
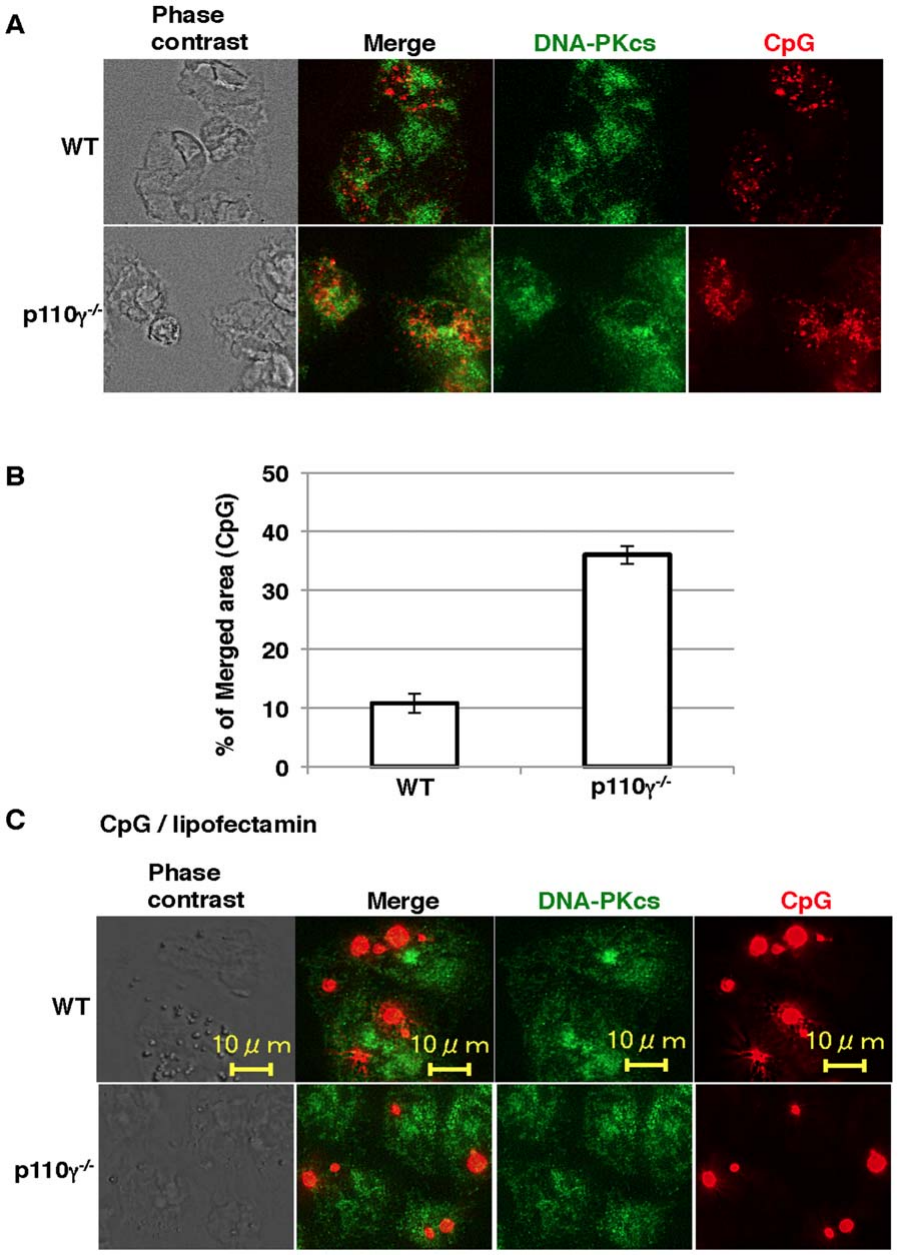
Increased co-localization of CpG and DNA-PKcs in p110γ^−/−^ cells. Macrophages from wild type or p110γ^−/−^ mice were incubated with 0.5 µM rhodamine-CpG (A) or rhodamine-CpG/lipofectamine LTX/Plus (C) for 60 min. The cells were washed, fixed, permeabilized, and stained with anti-DNA-PKcs antibody and Alexa 488-labeled secondary antibody. (B) The imaging data in (A) were quantified and shown as means ± SD.

CpG is internalized via endocytosis and immediately moves into the lysosomal compartment [5], [18], [19]. A recent study reported that endosomal CpG preferentially induces IL-12 production, but, when released from the endosome, it associates with DNA-PKcs in cytoplasm and induces a greater amount of IL-10 [7]. Therefore, we hypothesized that CpG localized to the endosomal compartment in the wild type cells, and was more efficiently released into the cytosol in p110γ^−/−^ cells. To quantify the co-localization of CpG and DNA-PKcs, macrophages were incubated with rhodamine-labeled CpG, fixed with formaldehyde, permeabilized, and incubated with anti-DNA-PKcs antibody. The merged area was calculated from the imaging data as described under materials and methods. The Co-localization area of CpG and DNA-PKcs was significantly increased in 110γ^−/−^ cells (Fig. 5A, B). Interestingly, CpG complexed with cationic liposomes composed of Lipofectamine (LTX) and Plus reagent localized in large vesicles both in wild type and p110γ^−/−^ cells, and scarcely co-localized with DNA-PKcs (Fig. 5C). Since wortmannin did not affect CpG uptake or localization of CpG or DNA-PKcs (data not shown), the PI3K inhibitor exclusively inhibits the kinase activity of DNA-PKcs.

### Manipulation of CpG localization with cationic liposomes abolished the effect of p110γ deficiency on cytokine production

**Figure 6:**
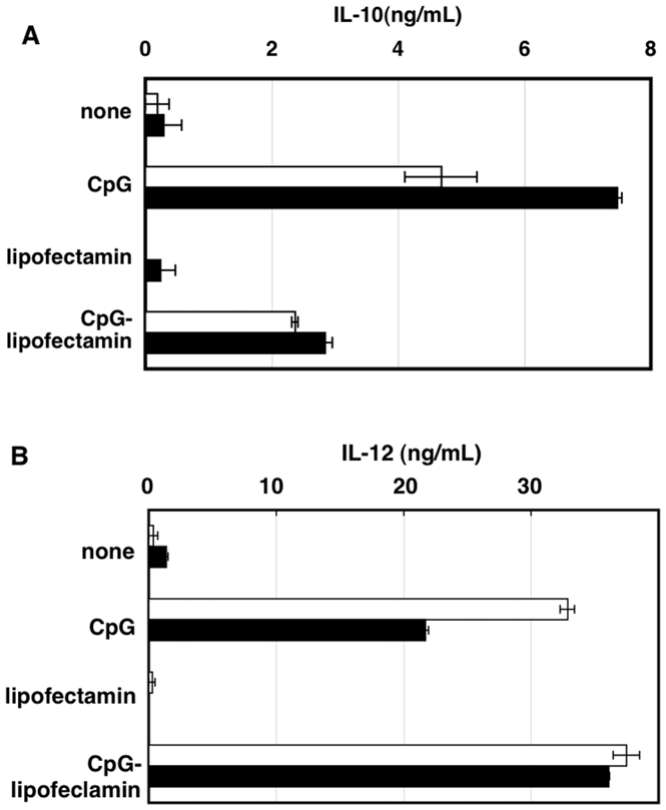
The effect of lipofection reagent on CpG-induced cytokine production. Macrophages from wild type (open bar) or p110γ^−/−^ mice (solid bar) were incubated in 24-well plates with 200 ng/mL CpG or CpG/lipofectamine LTX/Plus reagent complex for 18 h. The amount of IL-10 (A) and IL-12 (B) in the medium was determined by ELISA. The values are the means ± SD of duplicate cultures from three independent experiments.

It seemed interesting to determine IL-10 production by CpG complexed with the lipofection reagent, which hardly co-localizes with DNA-PKcs. When cells were stimulated with this CpG/lipofection reagent, IL-10 production was decreased both in wild-type and p110γ^−/−^ cells (Fig. 6). In addition, the augmentation of IL-10 production seen in p110γ^−/−^ cells was completely abolished using this delivery system (Fig. 6).

### CpG preferentially localized in the early endosome in wild type cells, and in lysosomes in p110γ^−/−^ cells

**Figure 7:**
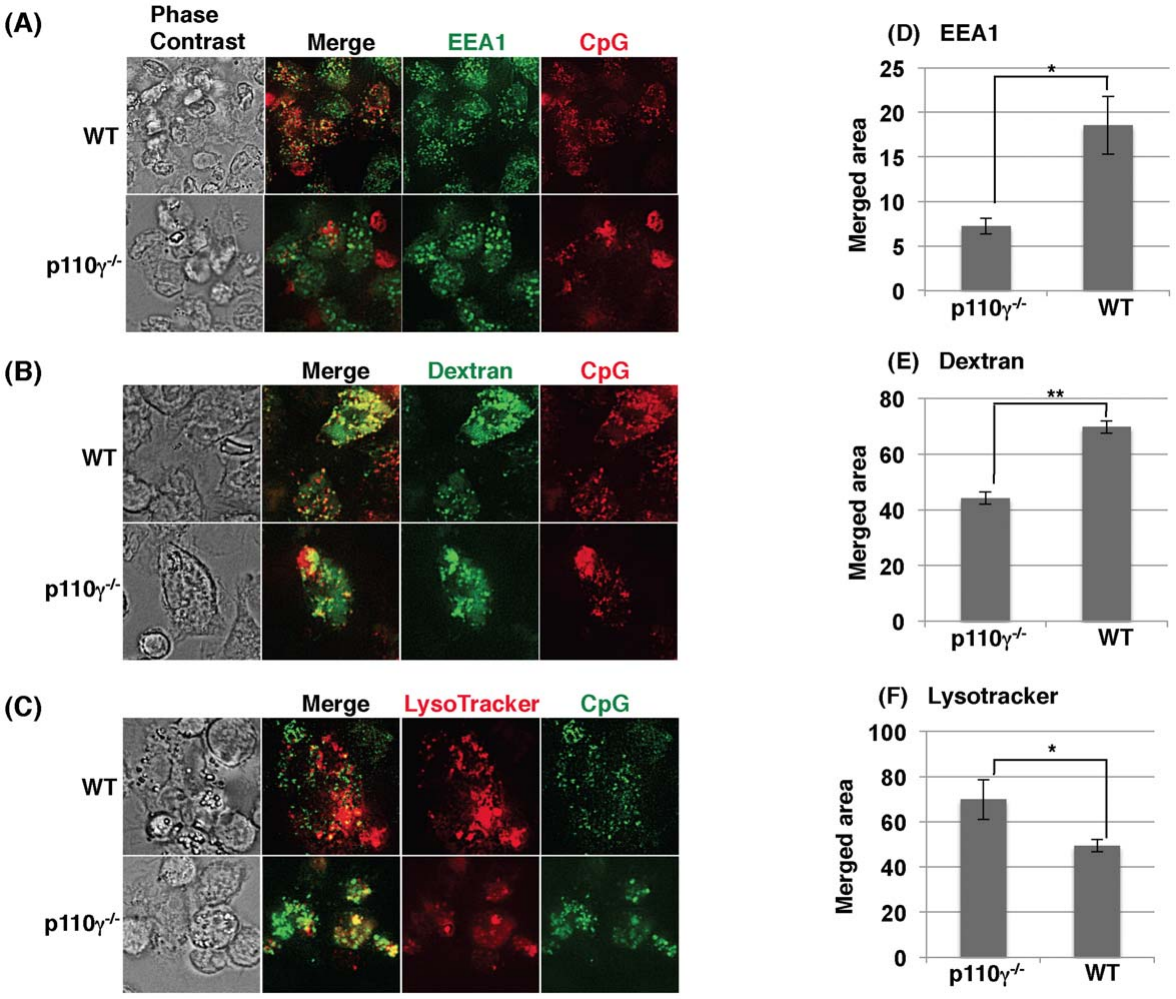
CpG localization in macrophages from wild type and p110γ^−/−^ mice. Macrophages from wild type or p110γ^−/−^ mice were incubated with 0.5 µM rhodamine-CpG (A, B) or FITC-CpG (C) for 30 min. FITC-dextran (1 mg/ml) (B) or LysoTracker red (50 nM) (C) was added simultaneously with CpG. The cells were washed and observed as live cells (B and C). In (A), the cells were fixed, permeabilized and stained with anti-EEA1 antibody and Alexa 488-labeled secondary antibody. The imaging data in (A, B, C) were quantified and shown as means ± SD in (D, E, F). *; p<0.05, **; p<0.01.

**Figure 8:**
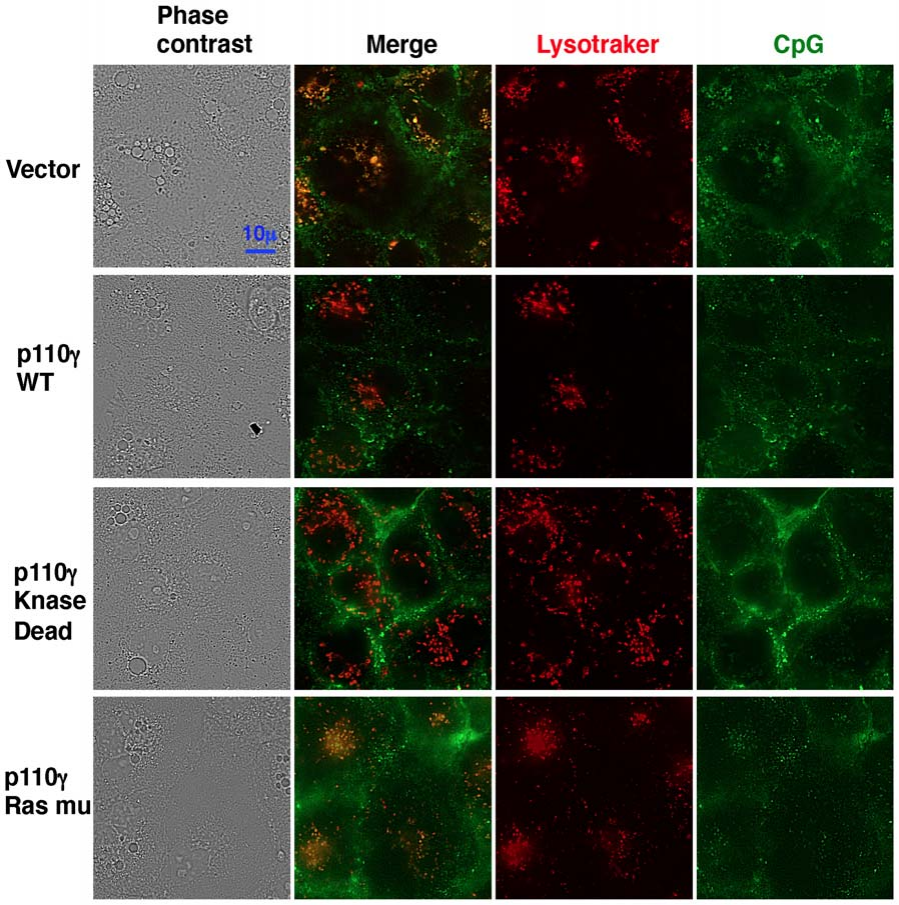
Effect of overexpression of p110γ on CpG localization in Cos7 cells. Cos7 cells were transfected with PI3K p110γor its mutant forms as described under materials and methods. Cells prepared in this manner were incubated with 50 nM LysoTracker red and 0.5 µM FITC-CpG for 30 min. The cells were washed and observed as live cells.

We next tested the cellular delivery of CpG using an early endosome marker, EEA1, an endosome marker, dextran, and an acidic lysosome marker, LysoTracker. Quantitative analysis of microscopic images showed that more CpG merged with EEA1 and dextran in wild type cells than in p110γ^−/−^ cells (Fig. 7A, B, D, E). By contrast, CpG preferentially merged with LysoTracker in p110γ^−/−^ cells more than in wild-type cells (Fig. 7C, F). To further investigate the role of p110γ in CpG localization, Cos7 cells were transfected with p110γ and its mutant forms (unlike macrophages, Cos7 cells do not express p110γ). As shown in Fig. 8, most of the CpG was co-localized with LysoTracker in Cos7 cells transfected with vehicle alone, while scarcely co-localized with LysoTracker in the cells transfected with wild type p110γ. Interestingly, overexpression of a kinase-dead mutant of p110γ also inhibited the acidification of CpG-containing endosome. By contrast, the Ras-binding domain mutant form showed no effect on the CpG localization. These results suggest that PI3K p110γ play a role in endosomal acidification independent of its kinase activity. Since endosomal acidification is known to precede the endosomal leakage, acidification of CpG containing endosome may accelerate CpG translocation to the cytosol and the resultant association with DNA-PKcs to increase IL-10 production in macrophages.

### CpG-induced but not LPS-induced IL-10 production was suppressed by inhibitors of endosomal acidification

**Figure 9:**
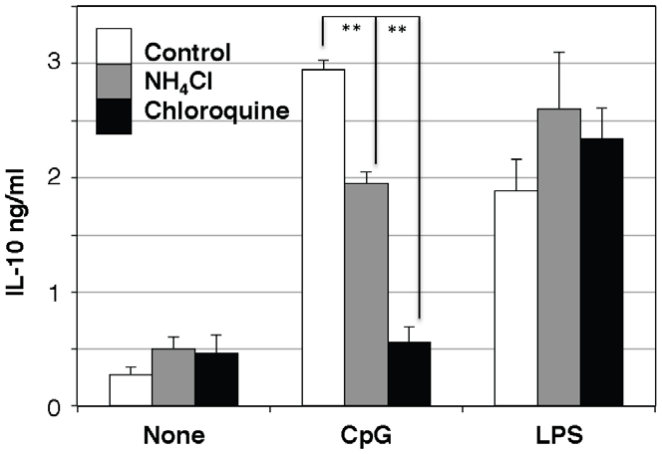
Effect of inhibitors of lysosomal acidification on IL-10 production. Macrophages from wild type mice were preincubated with 20 mM NH_4_Cl, 50 µM Chloroquine or vehicle for 15 min, followed by the addition of 10 ng/mL LPS or 200 ng/mL CpG, for 18 h. The amount of IL-10 in the medium was determined by ELISA. The values are the means ± SD of duplicate cultures from three independent experiments. **; p<0.01.

Effect of chemical inhibitors of endosomal acidification [20] on CpG-induced IL-10 production was next examined. Both NH_4_Cl and chloroquine strongly inhibited CpG-induced IL-10 production without affecting LPS-induced one (Fig. 9). The result supported our hypothesis that endosomal acidification is required for CpG-induced IL-10 production.

## Discussion

In this study, we have identified a novel function of PI3K p110γ in the regulation of CpG localization. We have demonstrated this function using p110γ^−/−^ macrophages and Cos7 cells transfected with p110γ. In macrophages, more CpG merged with the endosome markers, EEA1 and dextran, in wild type cells than in p110γ^−/−^ cells, whereas preferentially merged with the acidic lysosome marker, LysoTracker, in p110γ^−/−^ cells to a greater extent than in wild-type cells. In Cos7 cells, which do not express p110γ, most of the CpG was co-localized with LysoTracker, and scarcely co-localized with the dye in the cells transfected with p110γ. Another novel finding reported in this paper is that IL-10 production was increased specifically in p110γ^−/−^ cells following CpG-stimulation. In p110γ^−/−^ cells, the increased acidification of CpG containing endosomes and the resultant leakage of CpG to the cytosol, where DNA-PKcs resides, appears to be responsible for the modulation of cytokine production. For this reason, CpG-induced, but not LPS-induced, IL-10 production was almost completely abolished in SCID mice having mutations in DNA-PKcs. Furthermore, wortmannin, an inhibitor of DNA-PKcs, inhibited completely CpG-induced IL-10 production both in wild-type and in p110γ^−/−^ cells. In addition to these, manipulation of the delivery system with cationic liposomes, which severely blocked the cytosolic delivery of CpG both in p110γ^−/−^ and wild type cells, resulted in decreased IL-10 production. Finally, an intriguing point in this study is that the actions of p110γ on both the CpG delivery system and cytokine production were independent of its kinase activity.

Several kinase-independent functions of p110γ have been reported previously. Protein complexes containing p110γ are known to activate phosphodiesterase (PDE3B) in cardiomyocytes to degrade cAMP in a manner independent of its kinase activity [21], [22]. Since increases in the cAMP level results in augmentation of TLR-mediated IL-12 production, with a decrease in IL-10 production [23], we had hypothesized that the increased IL-10 production in the absence of p110γ might be the result of elevated cAMP levels. To answer this question we tested some reagents known to increase cAMP, such as forskolin, prostaglandin E_2_, 3-isobutyl-1-methylxanthin or dibutyryl cAMP in IL-10 production. Although these reagents more or less enhanced IL-10 production, the effect was not specific to CpG stimulation, but rather, was commonly observed in TLR-stimulation (data not shown). The other kinase-independent functions of p110γ are reported in vascular repair and platelet aggregation [22]. In addition, wild-type or the kinase-dead mutant of p110γ can block the growth of human colon cancer cells [24]. Although the mechanism of these kinase-independent actions of p110γ remains to be clarified, a scaffolding role for p110γ has been suggested [22].

p110γ^−/−^ mice show severe defects in immune responses, and are protected completely against systemic anaphylaxis [25]–[28]. Additionally, in models of rheumatoid arthritis, systemic lupus erythematosus, and atherosclerosis, loss of p110γ activity results in protection against disease progression [29]–[31]. Since IL-10 is an anti-inflammatory cytokine, the increased IL-10 production in p110γ-deficient cells may contribute, at least in a part, to protection against excessive inflammation. It is also likely that the increased IL-10 and decreased IL-12 production in p110γ^−/−^ macrophage might explain partly the development of colorectal carcinomas in p110γ^−/−^ mice [24], because the IL-12-mediated Th1 response favors effective anti-tumor immune responses [32]. Although further studies are needed to confirm the *in vivo* effect of p110γ on the translocation of CpG, our findings suggest that when p110γ is considered as a drug target for immune diseases [15], [33], [34], not only its lipid kinase function, but also its kinase-independent function should be considered.

## Materials and Methods

### Reagents

LPS (*E. coli* serotype 0111: B4), FITC-Dextran (average MW 40 kD) and C5a were from Sigma-Aldrich. Wortmannin was from Kyowa Medex (Tokyo, Japan). 5′-rhodamine-labeled, 5′-FITC-labeled and unlabeled CpG DNA (HPLC-purified phosphorothioate with the sequence of TCC ATG ACG TTC CTG ATG CT) were synthesized by Hokkaido System Science (Sapporo, Japan). LysoTracker Red was obtained from Lonza. Lipofectamine LTX Reagent, Plus Reagent and RPMI 1640 medium were from Invitrogen. The Protein Assay Kit was purchased from Bio-Rad. AS-252424 was from Cayman. Anti-pAkt (Ser473) antibody was from Cell Signalling, anti-Akt1/2 and anti-DNA-PKcs antibodies were from Santa Cruz, and anti-EEA1 was from GenScript. The IL-10 ELISA assay kit was from Biolegend.

### Animals and cell isolation

All animal experiments were carried out in accordance with the NIH Guide for Care and Use of Laboratory Animals and approved by the animal care and use committee at Hiroshima University (Permit number: A08-23 and A08-46).

Female C57BL/6 mice, 8–12 weeks old, were purchased from Japan SLC, Inc. SCID, C.B-17/lcr^+/+^, C.B-17/lcr-SCID/SCID mice were purchased from CLEA Japan. p110γ^−/−^ mice on the C57BL/6 background were bred and maintained at Akita University (Akita, Japan). Thioglycollate-elicited macrophages were harvested from these mice. Briefly, mice were injected intra-peritoneally with 2 mL 3% thioglycollate broth. After 3 days, the peritoneal exudate cells were collected by washing the peritoneal cavity with ice-cold phosphate-buffered saline (PBS). The cells were seeded at about 5–10×10^5^ cells/well in 24-well plates and incubated in humidified 5% CO_2_ at 37°C for 1–2 h in RPMI 1640 medium supplemented with 10% fetal bovine serum (FBS) (MBL, Nagoya, Japan), to allow the cells to adhere to the wells. Non-adherent cells were removed by washing with PBS and the attached cells were used for experiments. Cos7 cells [35] were cultured in DMEM medium supplemented with 10% FBS.

### Plasmids and transfection

Mammalian expression plasmids, pcDNA3 (Invitrogen), pcDNA3 containing wild-type p110γ, pcDNA3 containing a kinase-dead (R947P) mutant, or encoding a Ras binding site mutant (DASAA; T232D, K251A, K254S, K255A and K256A) [36] are transfected in to Cos7 cells using the Lipofectamine 2000 Reagent for 24 h.

### Western blot

Cells were washed with PBS and lysed in 50 µL lysis buffer containing 25 mM Tris-HCl (pH 7.4), 0.5% Nonidet P-40, 150 mM NaCl, 1 mM sodium orthovanadate (Na_3_VO_4_), 1 mM EDTA, 0.1% BSA, 20 mM sodium fluoride, 1 mM phenylmethylsulfonyl fluoride, 2 µM leupeptin, 20 µM p-amidinophenylmethylsulfonyl fluoride, and 1 mM dithiothreitol. The cell lysates were centrifuged at 15,000 rpm for 10 min. Supernatants were collected and the protein concentration was determined using the Bio-Rad assay kit. Total cell lysates (100 µg protein) were mixed with 10 µL 5× sample buffer (62.5 mM Tris, pH 6.8, 1% SDS, 10% glycerol, 5% 2-mercaptoethanol, and 0.02% bromophenol blue) and heated at 100°C for 5 min. The proteins were separated by SDS-PAGE and transferred electrophoretically onto a polyvinylidene difluoride (PVDF) membrane (Millipore). The membrane was blocked with 5% skim milk and incubated with the appropriate antibodies. Antibody binding was detected using a chemiluminescent substrate (Perkin-Elmer).

### ELISA

Macrophage culture supernatants were used for the quantification of p40 and IL-10 using a commercially available ELISA kit.

### Microscopy

Macrophages in multi-well, glass-bottom dishes (Greiner bio-one) were allowed to adhere for 60 min before the addition of rhodamine- or FITC-CpG. Where indicated, FITC-dextran or LysoTracker Red was added with CpG. The cells were washed four times to remove excess CpG before live cell imaging. Alternatively, the cells were fixed with 4% formaldehyde in PBS for 15 min, permeabilized with PBS containing 0.3% Triton X-100 and 0.5% BSA for 60 min, and incubated with anti-DNA-PKcs or anti-EEA1 at 4°C overnight. They were then incubated with Alexa 488-labeled goat anti-mouse IgG antibody (Fab')_2_ for 2 h at room temperature. Cos7 cells were seeded into the culture wells a day before the transfection. The cells were incubated with LysoTracker red and FITC-CpG for 15 min as indicated. The cells were washed four times with PBS before imaging. Microscopic studies were performed using the Keyence BZ-9000 (Keyence, Osaka, Japan). The imaging data were analyzed by the BZ-H2A application. Values of (merged area)/(CpG area)×100 were determined from at least 5 imaging data, and the data are shown as the means ± SD. Statistical differences were determined at the level of p<0.05 or 0.01 with Student's t test.
